# Supplementary material for: Investigation of the Antifungal and Anti-Aflatoxigenic Potential of Plant-Based Essential Oils against Aspergillus flavus in Peanuts
Source: J Fungi (Basel). 2020 Dec 21;6(4):383. doi: 10.3390/jof6040383 (PMC7767372; doi:10.3390/jof6040383)
Supplement: Supplementary file 1 [file jof-06-00383-s001.pdf]

Journal of *Fungi*

MDPI

Supplementary Materials: Investigation of the Antifungal and Anti-Aflatoxigenic Potential of Plant-Based Essential Oils against *Aspergillus flavus* in Peanuts

Table 1. (**a**) Raw data used to generate the calibration equation shown in Figure S1 below; Stock reference standard was 1µg/mL Aflatoxin B1 (Sigma Aldrich, St. Louis, MO, USA). (**b**) Raw data for sample analysis. Control = not Clove Oil treated. Column four was calculated based on the calibration equation above.

	**(a)**		
**Solution**	**µL Standard**	**ppm AFB1**	**Area Count**
1	100	10	1972962
2	200	20	3257785
3	400	40	6744612
4	500	50	8717443

	**(b)**		
**Sample**	**ppm Clove Oil**	**Area Count**	**ppm/g AFB1**
Control	0	6237366	29.97
Oil_500	500	5506910	26.18
Oil_1000	1000	4493832	21.00
Oil_1500	1500	3254477	19.64
Oil_2000	2000	3494229	10.64
Oil_2500	2500	4714857	12.50

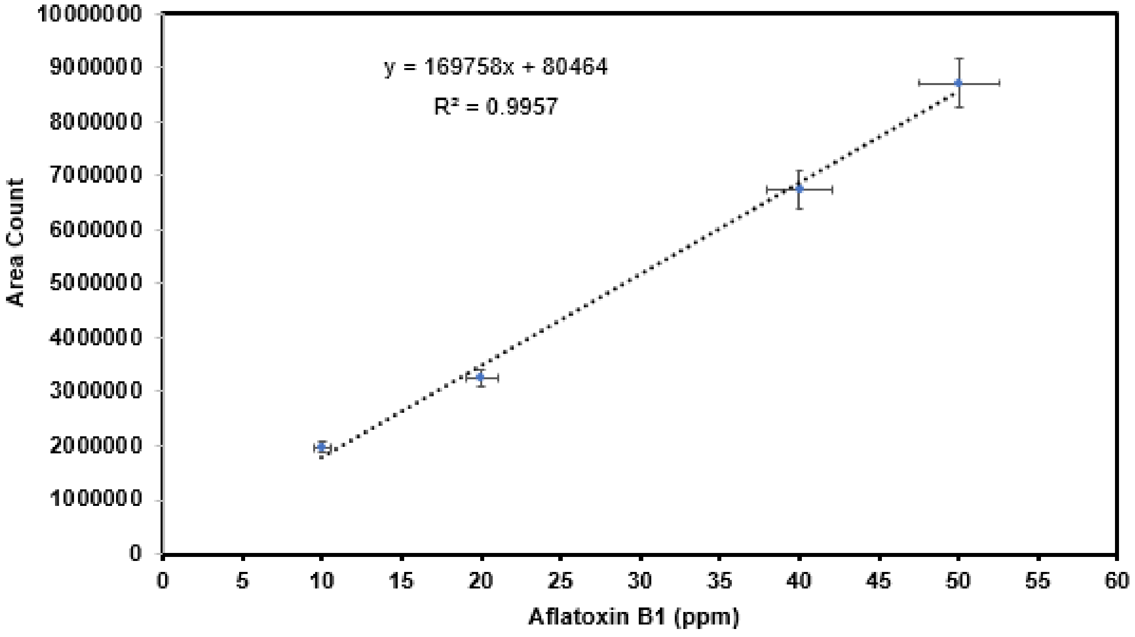

Figure S1. Calibration equation generated by linear regression from Table **S1** data.
